# Assessment of the Association of Vitamin D and the Risk of Tuberculosis among End-Stage Kidney Disease Population

**DOI:** 10.3390/life12111881

**Published:** 2022-11-14

**Authors:** Sithembiso Tiyandza Dlamini, Kyaw Moe Htet, Ei Chue Chue Theint, Wei-Ming Li, Hsin-Wen Chang, Hung-Pin Tu

**Affiliations:** 1 Graduate Institute of Medicine, College of Medicine, Kaohsiung Medical University, Kaohsiung 80708, Taiwan; 2 Department of Urology, Kaohsiung Medical University Hospital, Kaohsiung 80708, Taiwan; 3 Department of Urology, School of Medicine, College of Medicine, Kaohsiung Medical University, Kaohsiung 80708, Taiwan; 4 Department of Urology, Ministry of Health and Welfare, Pingtung Hospital, Pingtung 900, Taiwan; 5 Department of Applied Psychology, Hsuan Chuang University, 48 Hsuan Chuang Rd., Hsinchu City 30092, Taiwan; 6 Center for General Education, Hsuan Chuang University, Hsinchu City 30092, Taiwan; 7 Department of Public Health and Environmental Medicine, School of Medicine, College of Medicine, Kaohsiung Medical University, 100 Shih-Chuan 1st Road, Kaohsiung 80708, Taiwan; 8 Department of Medical Research, Kaohsiung Medical University Hospital, Kaohsiung 80708, Taiwan

**Keywords:** retrospective cohort study, kidney disease, end-stage kidney disease, vitamin D, tuberculosis

## Abstract

We investigated the role of vitamin D in the risk of tuberculosis (TB) among patients with end-stage kidney disease (ESKD). The retrospective cohort was conducted with data of 20,985 patients with kidney disease and 20,985 controls without kidney disease (1:1 matching on age of cohort entry and sex) in the duration of 1997–2010 from the Taiwan National Health insurance database. Then, by a case–cohort study, among 20,985 kidney disease, 3194 ESKD patients were identified with matched 3194 non-ESKD patients. Multivariate analyses revealed a significant association between kidney disease and tuberculosis (adjusted incidence rate ratio (IRR) 1.57 (1.33–1.86)), and the risk increased after 3 years of follow-up the (adjusted IRR 3.79 (2.55–5.62)), but after more years of follow-up no significance was observed. We also found that ESKD increases the risk of tuberculosis (adjusted IRR 3.67 (2.27–5.93)). However, vitamin D usage was not related with the tuberculosis risk in ESKD patients (*p* > 0.1783). Our study showed increased risk of tuberculosis in kidney disease and ESKD patients, and vitamin D was not beneficial in ESKD.

## 1. Introduction

End-stage kidney disease (ESKD) inflicts significant health and economic burdens on both individuals and the public population [1]. Chronic kidney disease (CKD) is a structural and functional progressive kidney damage lasting for more than 3 months. When kidney disease reaches the end stage, that is, stage 4 and 5, high levels of fluid, electrolytes and wastes products can build up in the body; this might cause severe decline of GFR over the periods of months or years [2]. The symptoms of worsening kidney function might include leg swelling, vomiting and confusion and reduced appetite; however, this disease can complicate to hypertension, heart failure, bone disease and anemia. CKD has since been an increasing endemic, with a worldwide prevalence of 8% to 16% [3]. Taiwan has the highest prevalence of CKD, and the reported prevalence of ESKD is 6.9% in adult populations in Taiwan [4]. The major causes of CKD in Taiwan are older age, diabetes, hypertension, smoking, obesity, regular use of herbal medicine, chronic lead exposure and hepatitis C, chronic glomerulonephritis and chronic interstitial nephritis [5]. There has since been a gap on the findings about the relationship between CKD and pulmonary TB. Not many studies have reported on the high prevalence of TB among patients with CKD.

According to the World Health Organization (WHO), tuberculosis (TB) remains one of the most leading infectious diseases in the world. The World Health Organization estimates 10.4 million new TB cases worldwide and 1.3 million related deaths in 2016. The relationship between active tuberculosis (TB) and chronic kidney disease (CKD) was first reported in a 1974 case reported on a dialysis patient. According to the National Institute for Health and Care Excellence guidelines, the relative risk for developing active TB is 10% to 25% in patients with CKD at any stage [6]. This relationship has since been an emerging global syndemic. There is a hypothesis that CKD may increase the risk of developing TB and related immunosuppression, especially in kidney transplants recipients. Due to this fact, diagnosis may be challenging because of the nonspecific symptoms, which are the same as extrapulmonary TB and peritoneal disease in patients receiving renal replacement therapy. This circumstance may cause TB diagnosis in dialysis patients to be delayed because of the extrapulmonary manifestations. Initially, it was believed that hemodialysis patients have a higher incidence of latent tuberculosis than peritoneal dialysis. Studies have argued this phenomenon is caused by the fact that hemodialysis patients have more frequent hospital visits and longer hospital stays. A study in Pakistan reported that low vitamin D levels were associated with a five-fold increased risk for progression to tuberculosis [7]. Given the rising scientific evidence regarding vitamin D’s multisystem role, the association between chronic kidney disease [8] with the loss of functional renal function to convert 25(OH)D to 1,25(OH)2D, calcitriol is reduced leading to a decline in plasma 1,25(OH)2D [9]. Vitamin D status in CKD patients is most commonly assessed on the basis of the plasma concentration of 25-hydroxyvitamin D or calcidiol. Vitamin D deficiency thresholds for population health are defined as 25(OH)D serum levels  <  25 or 30 nmol/L [10,11] The effects were shown to depend on the stage of the disease.

Observational studies in patients treated with hemodialysis showed that the use of active vitamin D sterols was associated with lower risk of all-cause mortality, regardless of parathyroid hormone levels [12]. Vitamin D supplementation in CKD is to prevent and treat the complications associated with secondary hyperparathyroidism in ESKD [13]. However, in the Japan Dialysis Active Vitamin D (J-DAVID) trial, treatment with alfacalcidol did not reduce the risk of composite cardiovascular events or the risk of all-cause mortality in hemodialysis patients without secondary hyperparathyroidism [14]. Some studies have conducted research on the effectiveness of vitamin D supplementation on plasma parathormone (PTH) concentrations, and it has been discovered to reduce the PTH concentrations. Some studies have argued that this effect depends on the patient’s characteristics [15], the dose–response of vitamin D supplementation and the response in PTH. The optimal concentration ranges of PTH and 25(OH)D for the management and prevention of CKD–MBD are not well established for each stage of CKD [16]. Some studies suggest that vitamin D supplementation, especially calcidiol, is not effective in CKD patients with hyperthyroidism due to the interacting of the medication cinacalcet, which reduces the parathyroid hormone (PTH) [16,17]. Studies have shown that there was a negative correlation between 25(OH)D and PTH levels [18].

Patients with ESKD are increasing, and they need dialysis; this is a major health problem because even TB is a commonly associated infectious disease. Many studies have argued that hemodialysis patients visit the hemodialysis room frequently, so they are more likely to acquire a Mycobacterium tuberculosis infection than peritoneal dialysis patients through airborne transmission in the HD rooms [19]. It is believed that vitamin D insufficiency arises at an early stage of the disease and tends to worsen with the progressive loss of renal function [20].

In this study, the main objective is to see through this research gap by conducting a cohort study using data from the National Health Insurance database in Taiwan to assess the relationship between patients with CKD and TB and more knowledge on the outcomes in patients taking vitamin D in ESKD renal patients.

## 2. Materials and Methods

### 2.1. Source of Data and Study Population

We conducted a nationwide, population-based retrospective cohort study to clarify the role of renal disease in the risk of tuberculosis, taking advantage of a well-established and large-size data set available from the Taiwan National Health Insurance Research Database (NHIRD). The Longitudinal Health Insurance Database 2010 (LHID2010) constitutes registration and claims data collected by the NHIRD program for a nationally representative group of 1 million individuals. The NHIRD contains all claims-related data, including patient personal information (e.g., age and gender), clinical diagnoses, prescribed medicines and health-care use. Disease diagnosis is assigned according to the International Classification of Diseases, 9th Revisions, and the Clinical Modification (ICD-9-CM) diagnostic codes. To protect patient confidentiality, all their identification numbers and medical institutions were encrypted before the release of the data for research purposes. Because no patient could be identified, informed consent was waived. The study protocol was reviewed and approved by the Institutional Review Committee of Kaohsiung Medical University Hospital (KMUHIRB-EXEMPT (I)-20190011), Taiwan.

**Figure 1:**
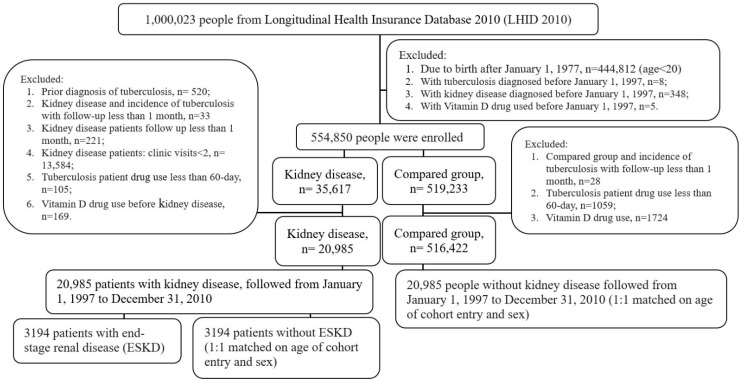
Flow chart for the selection of study patients.

The flowchart illustrating the selection process of participants is shown in Figure 1. From 1,000,023 people (LHID 2010), we excluded 445,173 people, due to birth after 1 January 1977 (age < 20) (*n* = 444,812), tuberculosis diagnosed before 1 January 1997 (*n* = 8), kidney disease diagnosed before 1 January 1997 (*n* = 348) and vitamin D used before 1 January 1997 (*n* = 5), respectively. A total of 554,850 people are enrolled with kidney disease (*n* = 35,617) and the compared group (*n* = 519,233). From the kidney disease patients, we excluded those with prior diagnosis of tuberculosis (*n* = 520), kidney disease and incidence of tuberculosis with follow-up of less than 1 month (*n* = 33), kidney disease patients with follow-up of less than 1 month (*n* = 221), kidney disease patients with clinic visits < 2 (*n* = 13,584), tuberculosis patients with drug use less than 60 days (*n* = 105) and vitamin D drug use before kidney disease (*n* = 169), respectively. From the compared group, incidence of tuberculosis with follow-up of less than 1 month (*n* = 28), tuberculosis patients with drug use of less than 60 days (*n* = 1059) and vitamin D drug use (*n* = 1724) were excluded. The final kidney disease patients (*n* = 20,985) were matched with the compared group (matched 1:1 on age of cohort entry and sex). Presence of kidney disease and tuberculosis were defined on the basis of the fulfillment of the following criteria: tuberculosis—3 or more clinic visits. The index date was the date of the first diagnosis of kidney disease. The main study outcome was TB diagnosed. We considered incident TB from the first kidney disease diagnosis to the date of TB diagnosis or the end of the study. The follow-up period began on 1 January 1997 and ended on the date of the first TB events, the end of the study (31 December 2010) or the end of the follow-up, whichever occurred first.

### 2.2. Ascertainment of Kidney Diseases and Tuberculosis

In total, 20,985 patients with kidney disease and 20,985 without kidney disease (matched 1:1 on age of cohort entry and sex) were included in this study. The outcome of interest was the documentation of tuberculosis by a physician. Assessment of renal disease (by ICD-9-CM code 403.01, 403.11, 403.91, 404.02, 404.03, 404.12, 404.13, 404.92, 404.93, 582.x, 583.0–583.7, 585.x, 586.x, 588.0, V42.0, V45.1, V56.x) was carried out during an outpatient or inpatient visit. Assessment of TB was conducted with ICD-9-CM codes of TB (010-018) plus the prescription of more than two anti-tuberculosis medications (i.e., isoniazid, rifampin, pyrazinamide, ethambutol, rifater, rifinah, streptomycin, cycloserine, prothionamide, amikacin, kanamycin, ciprofloxacin, moxifloxacin and levofloxacin) for more than 60 days. Subjects with kidney disease were followed from the index date to the date of first diagnosis of kidney disease. The ESKD patients were divided into hemodialysis (HD) and peritoneal dialysis (PD) cohorts according to the dialysis modalities with different operation codes (HD, 3995; PD, 5498), and the administration code was used to define renal dialysis (D8, hemodialysis and D9, peritoneal dialysis). The primary case definition of renal transplant recipients was having a physician-recorded primary diagnosis of a kidney replaced by transplant (ICD-9-CM V420) or having complications of the transplanted kidney (996.81) at either an outpatient or inpatient visit in NHIRD data sets. The ESKD patients receiving a prescription of vitamin D included alfacalcidol and calcitriol (Anatomical Therapeutic Chemical [ATC] code A1CC03 and A11CC04).

### 2.3. Comorbidities

In addition to the demographic risk factors of age, sex and region, we evaluated other potentially confounding factors for alcohol abuse, lipid disorders, obesity, hypertension, myocardial infarction, congestive heart failure, peripheral vascular disease, cerebrovascular disease, chronic pulmonary disease, rheumatologic disease, liver disease, diabetes mellitus and any malignancy. These comorbidities were diagnosed according to ICD-9-CM codes [21].

### 2.4. Statistical Analysis

A propensity analysis was performed to obtain a match of the propensity score for each patient with the covariates, such as age of cohort entry and sex. Continuous and categorical variables were analyzed using a t-test or Wilcoxon rank sum test and a chi-squared test, respectively, and the values obtained for the renal disease group and matched non-renal disease group were compared. The Kaplan–Meier method was used to estimate the survival curves for each group and the log-rank test was used to test for homogeneity among the survival curves. The incident rate ratio (IRR) was calculated using the PROC GENMOD generalized linear model to perform Poisson regression analysis, which is a log-linear model. Potential risk factors, such as comorbidities, were incorporated into the model. Significant results were those with *p* ≤ 0.05. All statistical analyses were performed using SAS statistical software (version 9.4, SAS Institute, Cary, NC, USA).

## 3. Results

Table 1 presents the demographic characteristics of the study population. This retrospective cohort study included 20,985 patients with chronic kidney disease and 20,985 with non-chronic kidney controls. In the 20,985 patients with renal disease, 2780 (13.2%) patients were undergoing hemodialysis only, and 291 (1.4%) were undergoing both hemodialysis and peritoneal dialysis. In patients with kidney disease, 290 developed tuberculosis (incidence rate 2.05, 95% confidence interval (CI) 2.04–2.06 per 1000-person years), and 334 of the patients without kidney disease had TB (incidence rate 1.14 95% CI 1.14–1.15 per 1000-person years). In the study, sex and age were distributed equally between the CKD and non-CKD and were matched in the participant’s characteristics. Most of the participants lived in the northern region. A higher proportion of participants with CKD than without CKD were diagnosed with hypertension (72.8% vs. 47.6%). Those with lipid disorder were more prevalent in the group with CKD than in the group with non-CKD (10,780, 51.4%).

**Figure 2:**
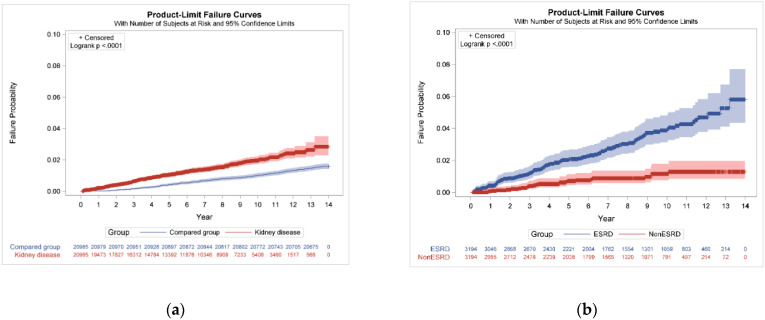
Cumulative incidence rates of tuberculosis: (**a**) with and without kidney disease; (**b**) with and without end-stage kidney disease (ESKD) in kidney disease patients.

Increased risk of development of tuberculosis was observed in our study (adjusted IRR 1.57 95% CI 1.33–1.86; Table 2). After 3 years follow-up, the risk of tuberculosis in chronic kidney disease was higher (adjusted IRR 3.79 (2.55–5.62)). However, after more years of follow-up, no significance was observed (adjusted IRR 1.33 95% CI 0.96–1.84; *p* = 0.0824). The Kaplan–Meier curves for the incidence of tuberculosis in individuals with and without kidney disease differed significantly (log-rank test *p* < 0.0001) (Figure 2a).

Table 3 presents the demographic characteristics of the ESKD population. This retrospective cohort study included 3194 patients with ESKD and 3194 non-ESKD. In the 3194 patients with ESKD, 2657 (83.2%) patients were undergoing hemodialysis only, 74 (2.3%) peritoneal dialysis, 271 (8.5%) both hemodialysis and peritoneal dialysis as well as 192 kidney transplant recipients (6.0%), respectively. In patients with ESKD, 98 developed tuberculosis (incidence rate 4.08, 95% confidence interval (CI) 4.03–4.13 per 1000-person years) and 25 of the patients without kidney disease had TB (incidence rate 1.17, 95% CI 1.16–1.19 per 1000-person years). We also found that ESKD increases the risk of tuberculosis (adjusted IRR 3.67 95% CI 2.27–5.93). The Kaplan–Meier curves for the incidence of tuberculosis in individuals with and without ESKD differed significantly (log-rank test *p* < 0.0001; Figure 2b).

**Figure 3:**
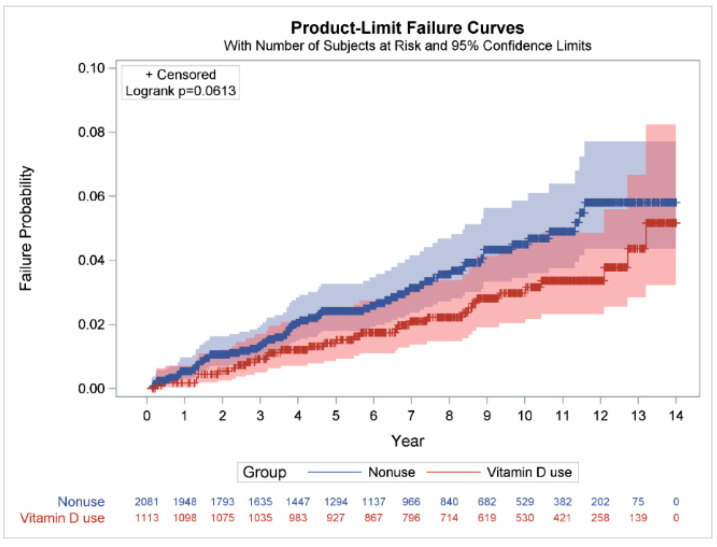
Cumulative incidence rates of tuberculosis in ESKD patients with and without vitamin D use. Differences in the cumulative incidence rates of tuberculosis in in ESKD patients with and without vitamin D use were compared using the log-rank test and Kaplan–Meier analysis. ESKD: end-stage kidney disease.

However, Vitamin D uses and dosages were not related with the reduced risk of tuberculosis in ESKD patients (Table 3). Vitamin D supplementation has no significance in ESKD (adjusted IRR 0.77, 95% CI 0.49–1.19; *p* = 0.2351). Moreover, we also observed that there was no dosage effect on ESKD patients (adjusted IRR > 0.56; *p* > 0.1783). Figure 3 shows the overall cumulative incidence of TB at the end of the follow-up period in ESKD patients with and without vitamin D use. The results show no significant differences in the cumulative incidence rate of tuberculosis between vitamin D use and non-use in the ESKD group log-rank test, *p* = 0.0613.

## 4. Discussion

This is a large cohort study conducted in 41,970 matched populations in Taiwan. The overall findings suggested that people with chronic kidney disease have a three-fold increased risk of developing tuberculosis compared with people without chronic kidney diseases. The important finding is that tuberculosis occurs early in chronic kidney disease patients, when at less than 3 years follow-up, the incident rate ratio of the patient with tuberculosis has already increased to (adjusted IRR 3.79 95% CI 2.55–5.62; the incident ratio of patients with tuberculosis gradually decreases in the longer follow-up year. Moreover, an increased risk of development of tuberculosis was observed in ESKD patients. Our results revealed that vitamin D uses and dosages were not related to the reduced risk of tuberculosis in ESKD patients, which is coincided withthe Lin et al. study [22]. The relationship between vitamin D level and TB seems to be controversial in ESKD at this current period, as some studies have stated that vitamin D has anti-mycobacterial activities [23]. Therefore, we need more molecular studies to evaluate the roles of vitamin D in the risk of TB.

In a study performed in Taiwan, the incidence rate of TB was similar in CKD stage 1 and stage 2 [24]. However, our findings may suggest that, indeed, the alteration in immunity during CKD may increase the risk of tuberculosis. A study also found that TB was significantly higher among those with CKD than among those without CKD [25]. This usually occurs in advance CKD patients because of the immunodeficiency status resulting from impaired T cells, B cell, neutrophils, monocytes and natural killer. The study results suggest that with the increasing severity of CKD, there is also a progressive increase in the risk of tuberculosis. There are many risk factors of tuberculosis that are also comorbidities of CKD that may also have increased TB infection in this cohort; these comorbidities may include diabetes and hypertension, which was found to be more prevalent in the CKD group than the non-CKD group. The association between diabetes mellitus and tuberculosis is mostly observed in countries where there is a high incidence of TB and the incidence of diabetes mellitus is also increasing.

However, there are still no studies to elucidate on the real mechanism of the effect of diabetes mellitus as a risk factor for TB. Some studies have suggested impaired cellular immunity, impairment of alveolar macrophages in the lungs, low levels of interferon gamma, pulmonary microangiopathy and micronutrient deficiency [26].

The number of participants on hemodialysis was high in the ESKD, which might increase the incidence of TB; previous studies have suggested that patients with chronic kidney disease and on dialysis are at risk of active tuberculosis (Table 2). A study performed in India suggested that TB in dialysis is associated with a poor prognosis and high mortality due to the delay in diagnosis and adverse effects of anti-TB drugs [27,28]. A study conducted in Northern Taiwan on patients with long-term dialysis had a hazard ratio of 2.041 [24]. Some also suggest that even after kidney transplantation, risk of tuberculosis is increased. Other cohort studies have found the IRR of TB to be 3.4 to 25.3 in dialysis patients compared to the general population. [29]. This is a very high risk on this patient, and they should be monitored closely for early diagnosis of tuberculosis. A study performed in Southern England found a higher incidence of TB in hemodialysis patients compared with patients on peritoneal dialysis. However, there were other studies that found the risk is not different from those with peritoneal dialysis [30].

This study also shows that vitamin D drug use in patients is not effective in reducing the incidence of tuberculosis in ESKD (Table 4).

Host genetic susceptibility has been suggested as one of the most important explanations for inter-individual differences in tuberculosis (TB) risk. The vitamin D receptor gene is located at chromosome 12 (12q13.11) [31]. Vitamin D can be obtained from nutrition, and it can also be synthesized by the human body during sunlight exposure. This form of vitamin D is biologically inactive, and then it is delivered into the liver, where it undergoes hydroxylation to become 25-hydroxy vitamin D. From the liver, it is taken to the kidneys, where it undergoes another hydroxylation to become 1α-25-dihydroxy vitamin D. This active form of vitamin D affects the immune function and regulates the activity of the defense immune system. During a tuberculosis infection, vitamin D binds to the vitamin D receptor gene in macrophages, and this binding activates synthesis of the antimicrobial peptide cathelicidin, which restricts M. tuberculosis intracellular growth in macrophages [32]. Vitamin D deficiency, which is commonly observed in ESKD [33], impairs monocyte function, reducing the production of cathelicidin, a peptide that is capable of destroying mycobacteria; hence, patients with CKD are given vitamin D supplementation to prevent the deficiency.. The main issue about the long-term use of vitamin D derivatives in patients with impaired renal function is the risk of inducing or accelerating the progression of renal failure [34]. Due to dietary restrictions in patients with ESKD on dialysis, and the presence of comorbidities that may result in longer hospitalization and less exposure to natural sunlight, CKD patients usually require vitamin D supplementation, especially cholecalciferol- and calcifediol-based supplements [33]. There is also a significant role in patients with lipid disorder, who seemed to also have a protective benefit from acquiring tuberculosis. The mechanism of these two is still unknown; however, some studies have suggested that host lipids act as a major source of carbon and energy for mycobacterium tuberculosis (M. tb), creating a favorable environment for it. They are important for the slow metabolizing population of the bacilli in the host [35]. Therefore, in our study, the findings suggest that the alteration in the lipids was beneficial for protection against M. TB survival in the host; however, the mechanism for this remains elusive and needs further investigations, since lipid disorders are supposed to make mycobacterium tuberculosis strive more in the host.

The strength of this study was that it was performed using data from a large population-based database in Taiwan. Therefore, the findings are likely applicable to the general population. However, electronic health databases with coding errors could be a problem of NHIRD. Health-care providers might resort to upcoding the diagnoses to more severe ones. Misclassification bias may thus become an issue if those diagnosis codes have not been properly validated. Although large data sets could potentially overcome this problem, the real impact of the incorrect coding awaits further elucidation [36].

A limitation of our study is that database-driven studies have greater potential for bias in observational studies. If we had combined multiple databases, the researcher would have been able to access more variables than those available in the NHIRD, such as physical examination results, laboratory data, stage of cancer, level of disability, quality of life, body mass index, smoking, marital status, education and household income, which were not included in our study [36]. Other important information was also unable to be obtained, such as hours of sun exposure and use of sunscreen. Analysis from NHANES III (the Third National Health and Nutrition Examination Survey) reported an inverse association between BMI and 25(OH)D levels in persons with CKD [37]. In this study, laboratory data from our participants were not retrieved from the database, which may be of importance to validate the immunological status of our study participants. This study did not include biochemical indices of bone metabolism and markers of bone and mineral disorders in patients who were given vitamin D therapeutic intervention, which can affect the PTH [38]. However, in the case of the suboptimal vitamin D levels and vitamin D deficiency in ESKD, vitamin D supplementation will be essential for those patients.

## 5. Conclusions

The study findings were that patients have an increased risk of early development of tuberculosis in chronic kidney diseases. Importantly, the risk was more increased in end-stage kidney diseases (ESKD), and vitamin D supplementation use might not be helpful to reduce the incidence of tuberculosis in this group. This leaves a gap in the health system in the treatment guidelines of chronic kidney disease, and these patients should be screened routinely and carefully for early detection of TB infection. This study provides the current actual knowledge and addresses the questions regarding vitamin D supplementation in ESKD, and its clinical benefit remains elusive in the scientific community.

## Figures and Tables

**Table 1:** Characteristics of patients with kidney disease and the comparison group.

	Kidney Disease	Compared Group	*p* Value
N	20,985	20,985	
Dialysis, *n* (%)			
Hemodialysis alone	2780 (13.2)	-	-
Peritoneal dialysis alone	80 (0.4)	-	-
Hemodialysis and peritoneal dialysis	291 (1.4)	-	-
Kidney transplant recipients	192 (0.9)	-	-
Tuberculosis *, *n* (%)	290 (1.4)	334 (1.6)	0.0760
Follow-up duration median (IQR), years	3.7 (1.7–6.6)	8.0 (4.9–11.2)	<0.0001
Follow-up duration groups, *n* (%)			
≤3 years	120 (41.4)	34 (10.2)	
>3 to 6 years	86 (29.7)	79 (23.7)	
>6 to 9 years	53 (18.3)	70 (21.0)	
>9 years	31 (10.7)	151 (45.2)	<0.0001
Age of cohort entry mean (SD), years	51.8 (14.2)	51.8 (14.2)	0.9501
Age group, *n* (%)			
20 to 30	1578 (7.5)	1578 (7.5)	
>30 to 40	3177 (15.1)	3177 (15.1)	
>40 to 50	4751 (22.6)	4751 (22.6)	
>50 to 60	4525 (21.6)	4525 (21.6)	
>60 to 70	4901 (23.4)	4901 (23.4)	
>70	2053 (9.8)	2053 (9.8)	1.0000
Sex, *n* (%)			
Males	11,242 (53.6)	11,242 (53.6)	
Females	9743 (46.4)	9743 (46.4)	1.0000
Residential region, *n* (%)			
Northern	9597 (45.7)	9748 (46.5)	
Central	4678 (22.3)	4899 (23.3)	
Southern	6038 (28.8)	5594 (26.7)	
Eastern and other region	672 (3.2)	744 (3.5)	<0.0001
Comorbidities, *n* (%)			
Alcohol abuse	404 (1.9)	214 (1.0)	<0.0001
Lipid disorders	10,780 (51.4)	5993 (28.6)	<0.0001
Obesity	306 (1.5)	149 (0.7)	<0.0001
Hypertension	15,274 (72.8)	9986 (47.6)	<0.0001
Myocardial infarction	380 (1.8)	176 (0.8)	<0.0001
Congestive heart failure	2709 (12.9)	1009 (4.8)	<0.0001
Peripheral vascular disease	1418 (6.8)	712 (3.4)	<0.0001
Cerebrovascular disease	3552 (16.9)	2253 (10.7)	<0.0001
Chronic pulmonary disease	5330 (25.4)	3859 (18.4)	<0.0001
Rheumatologic disease	1011 (4.8)	501 (2.4)	<0.0001
Liver disease	5083 (24.2)	2726 (13.0)	<0.0001
Diabetes mellitus	5286 (25.2)	2768 (13.2)	<0.0001
Any malignancy	1720 (8.2)	1061 (5.1)	<0.0001
Vitamin D drugs (ATC code), *n*(%)			
A11CC03 (alfacalcidol)	297 (1.4)	-	-
A11CC04 (calcitriol)	1138 (5.4)	-	-
A11CC03 or A11CC04	1306 (6.2)	-	-

IQR: interquartile range; SD: standard deviation; ATC: Anatomical Therapeutic Chemical Classification. Comorbidities were defined as more than two outpatient claims. Data of continuous and categorical variables were analyzed using the *t*-test or Wilcoxon rank sum test and chi-squared test to compare the data of kidney disease and the comparison group. * Active TB: ICD-9-CM codes of TB (010-018) plus the prescription of more than two anti-tuberculosis medications (i.e., isoniazid, rifampin, pyrazinamide, ethambutol, rifater, rifinah, streptomycin, cycloserine, protionamide, amikacin, kanamycin, ciprofloxacin, moxifloxacin and levofloxacin) for more than 60 days.

**Table 2:** Association of kidney disease with the risk of incident tuberculosis.

	Tuberculosis/ Total Subjects, %	Person–Years	Events Per 1000 Person–Years (95% CI)	IRR (95% CI)	*p* Value	Adjusted IRR (95% CI)	*p* Value
Compared group	334/20,985, 1.59	291,773.08	1.14 (1.14–1.15)	1.00		1.00	
Kidney disease	290/20,985, 1.38	141,549.53	2.05 (2.04–2.06)	1.79 (1.53–2.09)	<0.0001	1.57 (1.33–1.86)	<0.0001
Follow-up duration *							
≤3 years							
Compared group	34/20,985, 0.16	62,920.18	0.54 (0.54–0.54)	1.00		1.00	
Kidney disease	120/20,985, 0.57	56,041.95	2.14 (2.12–2.16)	3.96 (2.71–5.80)	<0.0001	3.79 (2.55–5.62)	<0.0001
>3 to 6 years							
Compared group	79/20,951, 0.38	125,588.8	0.63 (0.63–0.63)	1.00		1.00	
Kidney disease	86/16,303, 0.53	91,174.27	0.94 (0.94–0.95)	1.50 (1.10–2.04)	0.0093	1.33 (0.96–1.84)	0.0824

Incidence rate ratio (IRR) was calculated by using generalized linear model (PROC GENMOD) to perform Poisson regression analysis (a log-linear model). Adjusted IRRs were calculated after adjustment for residential region and comorbidities of alcohol abuse, lipid disorders, obesity, hypertension, myocardial infarction, congestive heart failure, peripheral vascular disease, cerebrovascular disease, chronic pulmonary disease, rheumatologic disease, liver disease, diabetes mellitus and any malignancy by using a cox proportional-hazards regression model. * For follow-up period analysis, kidney disease and risk of incident tuberculosis at the more than 3 to 6 years as excluded subjects of less than 3 years were calculated.

**Table 3:** Characteristics of end-stage kidney disease patients and kidney disease patients.

	ESKD	NonESKD	*p* Value
N	3194	3194	
Dialysis, *n* (%)			
Hemodialysis alone	2657 (83.2)	-	-
Peritoneal dialysis alone	74 (2.3)	-	-
Hemodialysis and peritoneal dialysis	271 (8.5)	-	-
Kidney transplant recipients	192 (6.0)	-	-
Tuberculosis, *n* (%)	98 (3.1)	25 (0.8)	<0.0001
Follow-up duration median (IQR), years	3.9 (1.7–7.4)	3.3 (2.4–5.3)	0.6085
Follow-up duration groups, *n* (%)			
≤3 years	36 (36.7)	11 (44.0)	
>3 to 6 years	26 (26.5)	8 (32.0)	
>6 to 9 years	24 (24.5)	3 (12.0)	
>9 years	12 (12.2)	3 (12.0)	0.5919
Age of cohort entry mean (SD), years	50.3 (13.1)	50.4 (13.3)	0.8923
Age group, *n* (%)			
20 to 30	199 (6.2)	199 (6.2)	
>30 to 40	519 (16.2)	519 (16.2)	
>40 to 50	908 (28.4)	908 (28.4)	
>50 to 60	726 (22.7)	726 (22.7)	
>60 to 70	629 (19.7)	629 (19.7)	
>70	213 (6.7)	213 (6.7)	1.0000
Sex, *n* (%)			
Males	1575 (49.3)	1575 (49.3)	
Females	1619 (50.7)	1619 (50.7)	1.0000
Residential region, *n* (%)			
Northern	1372 (43.0)	1476(46.2)	
Central	737 (23.1)	752(23.5)	
Southern	981 (30.7)	893(28)	
Eastern and other	104 (3.3)	73(2.3)	0.0037
Comorbidities, *n* (%)			
Alcohol abuse	39 (1.2)	64 (2.0)	0.0130
Lipid disorders	1648 (51.6)	1650 (51.7)	0.9601
Obesity	23 (0.7)	53 (1.7)	0.0005
Hypertension	2851 (89.3)	2172 (68.0)	<0.0001
Myocardial infarction	77 (2.4)	49 (1.5)	0.0118
Congestive heart failure	642 (20.1)	341 (10.7)	<0.0001
Peripheral vascular disease	282 (8.8)	174 (5.4)	<0.0001
Cerebrovascular disease	586 (18.3)	481 (15.1)	0.0004
Chronic pulmonary disease	683 (21.4)	751 (23.5)	0.0414
Rheumatologic disease	111 (3.5)	169 (5.3)	0.0004
Liver disease	668 (20.9)	780 (24.4)	0.0008
Diabetes mellitus	1048 (32.8)	725 (22.7)	<0.0001
Any malignancy	314 (9.8)	247 (7.7)	0.0031
Vitamin D drugs (ATC code), *n*(%)			
A11CC03 (alfacalcidol)	240 (7.5)	8 (0.3)	<0.0001
Patient visits median (IQR), frequency	6.5 (3.0–16.0)	4.0 (1.5–6.0)	0.0587
Total dose median (IQR)	51.9 (17.3–171.6)	29.0 (21.0–96.3)	0.3592
A11CC04 (calcitriol)	996 (31.2)	19 (0.6)	<0.0001
Patient visits median (IQR), *n*	9.0 (4.0–22.0)	6.0 (2.0–16.0)	0.1516
Total dose median (IQR)	54.1 (17.6–137.0)	38.5 (14.0–117.5)	0.5898
A11CC03 or A11CC04	1113 (34.8)	27 (0.8)	<0.0001
Patient visits median (IQR)	10.0 (4.0–23.0)	5.0 (2.0–11.0)	0.0061
Total dose median (IQR)	60.3 (18.8–152.1)	31.5 (14.0–117.5)	0.1975

ESKD: end-stage kidney disease; IQR: interquartile range; SD: standard deviation. Comorbidities were defined as more than three outpatient claims. Data of continuous and categorical variables were analyzed using the *t*-test or Wilcoxon rank sum test and chi-squared test to compare the data of ESKD and the non-ESKD patients.

**Table 4:** Vitamin D use did not reduce the incidence of tuberculosis risk in patients with end-stage kidney disease.

	Tuberculosis /Total Subjects, %	Person–Years	Events Per 1000 Person–Years (95% CI)	IRR (95% CI)	*p* Value	Adjusted IRR (95% CI)	*p* Value
ESKD							
None	25/3194, 0.78	21,330.40	1.17 (1.16–1.19)	1.00		1.00	
Yes	98/3194, 3.07	24,041.22	4.08 (4.03–4.13)	3.48 (2.24–5.40)	<0.0001	3.67 (2.27–5.93)	<0.0001
ESKD with vitamin D use							
Nonuse	66/2081, 3.17	14,006.34	4.71 (4.63–4.79)	1.00		1.00	
Use	32/1113, 2.88	10,034.88	3.19 (3.13–3.25)	0.68 (0.44–1.03)	0.0699	0.77 (0.49–1.19)	0.2351
Vitamin D use							
Nonuse	66/2081, 3.17	14,006.34	4.71 (4.63–4.79)	1.00			
0–6750	6/279, 2.15	2334.04	2.57 (2.47–2.68)	0.55 (0.24–1.26)	0.1553	0.56 (0.24–1.30)	0.1783
6751–21,690	8/275, 2.91	2304.91	3.47 (3.33–3.62)	0.74 (0.35–1.53)	0.4141	0.83 (0.39–1.74)	0.6183
21,691–54,810	8/281, 2.85	2663.54	3.00 (2.89–3.12)	0.64 (0.31–1.33)	0.2290	0.75 (0.36–1.58)	0.4520
>54,810	10/278, 3.60	2732.38	3.66 (3.53–3.80)	0.78 (0.40–1.51)	0.4564	0.95 (0.48–1.89)	0.8795

ESKD: end-stage kidney disease. Incidence rate ratio (IRR) was calculated by using generalized linear model (PROC GENMOD) to perform Poisson regression analysis (a log-linear model). Adjusted IRRs were calculated after adjusted covariates of residential region and comorbidities of alcohol abuse, lipid disorders, obesity, hypertension, myocardial infarction, congestive heart failure, peripheral vascular disease, cerebrovascular disease, chronic pulmonary disease, rheumatologic disease, liver disease, diabetes mellitus and any malignancy by using a generalized linear model. We classified the average vitamin D dose by using two approaches: stratifying the vitamin D exposure into yes or no and categorizing the per day milligram (mg) according to a quartile method. Daily exposure vitamin D of alfacalcidol and calcitriol dose: the accumulate vitamin D dose divided by the total follow-up days (by the first use vitamin D date until the index date of tuberculosis or to study end). The vitamin D dose–response (daily exposure dose) was analyzed after adjusted covariates using a by using generalized linear model.

## Data Availability

Not applicable.
